# A 3D Bioprinted Gut Anaerobic Model for Studying Bacteria–Host Interactions

**DOI:** 10.34133/research.0058

**Published:** 2023-02-27

**Authors:** Liqin Cheng, Tingting Liu, Qiongg Liu, Liming Lian, Guosheng Tang, Luis Santiago Mille, Fabricio Romero García, Lars Engstrand, Yu Shrike Zhang, Juan Du

**Affiliations:** ^1^ Centre for Translational Microbiome Research (CTMR), Department of Microbiology, Tumor, and Cell Biology, Karolinska Institutet, Stockholm, Sweden.; ^2^ Division of Engineering in Medicine, Department of Medicine, Brigham and Women’s Hospital, Harvard Medical School, 65 Landsdowne Street, Cambridge, MA 02139, USA.; ^3^ Science for Life Laboratory, Stockholm, Sweden.

## Abstract

The role of the human intestinal tract in host–microbe interactions has been highlighted in recent years. Several 3-dimensional (3D) models have been developed to reproduce the physiological characteristics of the human gut and to investigate the function of the gut microbiota. One challenge for 3D models is to recapitulate the low oxygen concentrations in the intestinal lumen. Moreover, most earlier 3D culture systems used a membrane to physically separate bacteria from the intestinal epithelium, which has sometimes made the studies of bacteria adhering to or invading cells less feasible. We report the establishment of a 3D gut epithelium model and cultured it at high cell viability under an anaerobic condition. We further cocultured intestinal bacteria including both commensal and pathogen directly with epithelial cells in the established 3D model under the anaerobic condition. We subsequently compared the gene expression differences of aerobic and anaerobic conditions for cell and bacterial growth via dual RNA sequencing. Our study provides a physiologically relevant 3D gut epithelium model that mimics the anaerobic condition in the intestinal lumen and supplies a powerful system for future in-depth gut–microbe interactional investigations.

## Introduction

The human gut is inhabited by trillions of microorganisms, which are important members of the human microbiome. A healthy and balanced human microbiome protects us from diseases and functions as a natural barrier against pathogens. Gut microbiome disbalances and the overgrowth of pathogenic bacteria have been linked to various disease conditions, such as infectious diseases, inflammatory bowel disease, and colorectal cancer [1–3]. *Salmonella* is one of the most common causes of diarrhea, inducing approximately 94 million cases of gastroenteritis and 155,000 deaths every year [4]. In contrast, *Lactobacillus* is one of the commensal gut microbiota that have been shown to play a protective role against the colonization of pathogens [5,6].

With the development of next-generation sequencing, DNA/RNA/protein sequencing had become powerful tools to study the interplay of gut pathogens and host [7–11]. Dual RNA sequencing (RNA-seq) studies both the pathogenic bacteria and the host cell simultaneously [6,12]. Earlier studies with dual RNA-seq found that different gene expressions of *Salmonella* impaired cell functions, modulated immune response, and changed the vesicle transport inside host cells [12–14]. However, there are only few studies with RNA-seq that compared the bacterial and host gene expression under different oxygenation conditions. In this study, we used dual RNA-seq to analyze the interaction between bacteria and host cells under both aerobic and anaerobic conditions.

Engineered 3-dimensional (3D) models become powerful tools to a reproduce the physiological characteristics of the human gut [15,16]. Human cells seeded into a 3D hydrogel have been shown to display a morphology characteristic of the human intestine and have been inoculated with bacteria to investigate the interactions between bacteria and host [17]. Moreover, the host–microbe interactions can be observed in real time and conveniently investigated through the injection of microbial strains into 3D models [18,19]. Furthermore, 3D tissue models may be inoculated with pathologies with high interindividual variations from patients to generate “living biobanks” for phenotypic or drug screens [20].

Among the various methods for generating the barrier tissue interfaces, sacrificial (bio)printing is perhaps one of the most suitable, as it enables the creation of embedded microchannel structures within extracellular matrix (ECM)-mimicking matrices for cell seeding, forming physiological configurations similar to those of their native microenvironments [21,22]. For example, we successfully established a ductal carcinoma-on-chip model in our former study using sacrificially printed hollow microchannels [23], among others [24–26]. Accordingly, we hypothesized that it would be possible to utilize a similar technique to produce a 3D model to mimic the in vivo-like condition of gut epithelial cells and investigate the gut–microbe interactions.

A potential challenge for 3D models, such as those for the intestine, is to recapitulate the low oxygen concentrations within the intestinal lumen even though the intestinal vascular network supplies ample oxygen for the epithelial cells [27]. Changes in the oxygen concentrations in the lumen of the human intestine would directly affect the composition and metabolism of gut microbiota and most intestinal bacteria, which are obligate anaerobes requiring oxygen concentrations below 0.5% [28]. Only few past anaerobic 3D culture systems used a nanoporous membrane or the membrane of the Transwell insert to physically separate the bacteria from the intestinal epithelium and thereby provide oxygen only for epithelial cells, creating low-oxygen environments for intestinal bacterial species [29–31]. However, these methods can sometimes limit the studies of the gut microbes that directly adhere to or invade epithelial cells, especially pathogens. Herein, we demonstrated the application of our sacrificially printed 3D gut model to investigate the direct interactions of *Salmonella* and *Lactobacillus* with epithelial cells and compared both host and bacteria gene expression changes between the anaerobic condition and the conventional aerobic condition.

## Results

### Sacrificial printing of microchannel-embedded hydrogel constructs

**Fig. 1.**
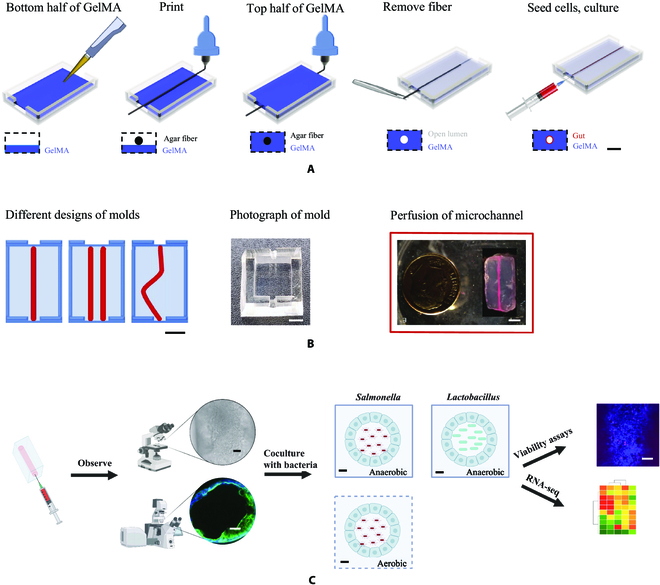
Sacrificial printing of hydrogel-embedded hollow microchannels. (A) Schematic view of the typical process of sacrificial printing: A layer of GelMA solution (half of the mold) is added to the bottom of the mold; after semi-crosslinking by UV for 10 s, an agar fiber is printed at the predefined location; the GelMA solution is further dispersed to fill the rest of the mold and crosslinked for 40 s; the agar fiber is removed from the surrounding crosslinked GelMA to allow for the formation of the microchannel; and cells are seeded on the interior surface of the microchannel and subjected to subsequent culture. (B) Schematics and photographs showing straight and curvy microchannels rapidly designed and fabricated using sacrificial printing: the different designs of microchannels, photograph of the mold, and perfusion of the different microchannels. (C) Schematic representation of the experimental workflow for utilizing the 3D hydrogel model to coculture cells and bacteria under aerobic and anaerobic conditions from seeding cells into the 3D hydrogel, observing cell growth and viability under binocular light microscope and confocal fluorescence microscope, and performing further gene expression comparison by RNA sequencing (RNA-seq). The scale bars in (A) and (B) are 2 cm, and the scale bars in (C) are 100 μm.

The typical process for sacrificially printing tissue-like constructs with embedded hollow microchannels is divided into 5 steps (Fig. 1A). In the first step, the polydimethylsiloxane (PDMS) mold is added with gelatin methacryloyl (GelMA) solution (the entire volume of which is half that of the mold) (Fig. 1A). After semi-crosslinking by ultraviolet (UV) light for 10 s, the agar fiber is printed at the predefined location (Fig. 1A). Then, the GelMA solution is dispersed to fill the rest of the mold and crosslinked for 40 s (Fig. 1A). The template and the agar fiber are gently removed from the surrounding GelMA ECM via physical force to allow the hollow microchannel to form (Fig. 1A). The microchannel is seeded with cells on the interior surface and cultured for subsequent use (Fig. 1A).

As shown in Fig. 1B, straight and curvy microchannels embedded within GelMA hydrogel constructs could be rapidly designed and sacrificially printed. These constructs could then be seeded with different types of cells for different intended biological applications. In this study, we used the 3D hydrogels to seed gut epithelial cells and studied their viability and gene expressions in coculture with *Salmonella* or *Lactobacillus* under aerobic and anaerobic conditions (Fig. 1C).

### Construction of the 3D gut model

**Fig. 2.**
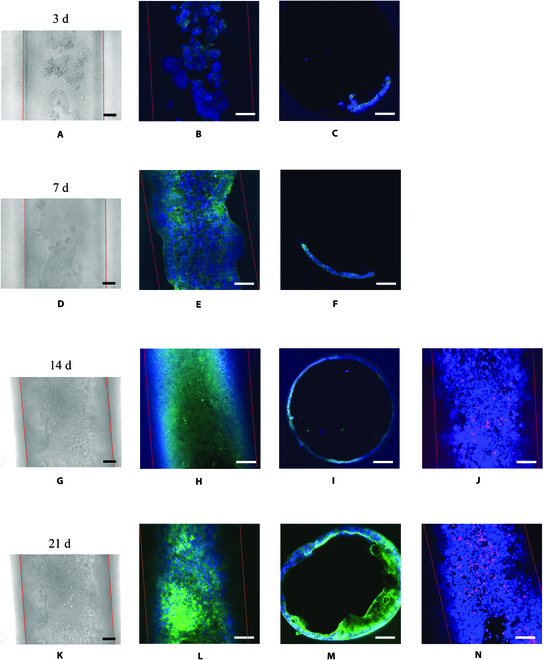
Optical micrographs showing the proliferation of the cells over a culture period of 21 d in the gut 3D model under binocular light microscope and confocal fluorescence microscope. Microchannel region populated by Caco-2 cells under binocular light microscope and stained for cytokeratin (green fluorescence) and nuclei (blue fluorescence Hoechst 33342 solution) at 3 d (A to C), 7 d (D to F), 14 d (G to I), and 21 d (K to M) of culture, respectively. The red dashed lines indicate the microchannel edge. Live/dead cells were also analyzed after 14 d (J) and 21 d (N) of culture. All the live cells were stained with blue fluorescence Hoechst 33342 solution. Only the dead cells were stained with red propidium iodide (PI) fluorescence. The scale bars are 100 μm.

The microchannels of the 3D hydrogels were seeded with Caco-2 cells and allowed the cells to grow for up to 3 weeks that we evaluated. The proliferation of the cells was followed and observed under bright-field microscopy and confocal fluorescence microscopy. The Caco-2 cells attached to the interior surface of the microchannels and were focally distributed in the microchannels 3 d after seeding (Fig. 2A to C). Observation of the Caco-2 cells under confocal microscopy with cells stained for pan-cytokeratin (green) and nuclei (blue) clearly showed that the cells attached and grew in the hydrogel 3 d after seeding (Fig. 2B). The cross section of the hydrogel-based model showed the cells covering a small area of the microchannel on day 3 (Fig. 2C). On day 7, the cells proliferated and merged, distributing at the bottom of the microchannel, which occupied approximately one-quarter of the microchannel (Fig. 2D to F). The cells covered the entire microchannel surface with a single layer in 2 weeks (Fig. 2G to I). Viability assays showed that most Caco-2 cells were alive in the microchannel after 14 d of culture (Fig. 2J). The cells continued proliferating after confluency and formed villi-like structures at 21 d, which could be observed through the cross section (Fig. 2K to M). The cells had grown inward the microchannel up to around 10 cell layers (Fig. S1). Viability assays showed that most Caco-2 cells remained alive in the microchannel after 21 d of culture (Fig. 2N).

### Cell gene expressions under the anaerobic condition did not notably change compared to the aerobic condition in the 3D gut model

**Fig. 3.**
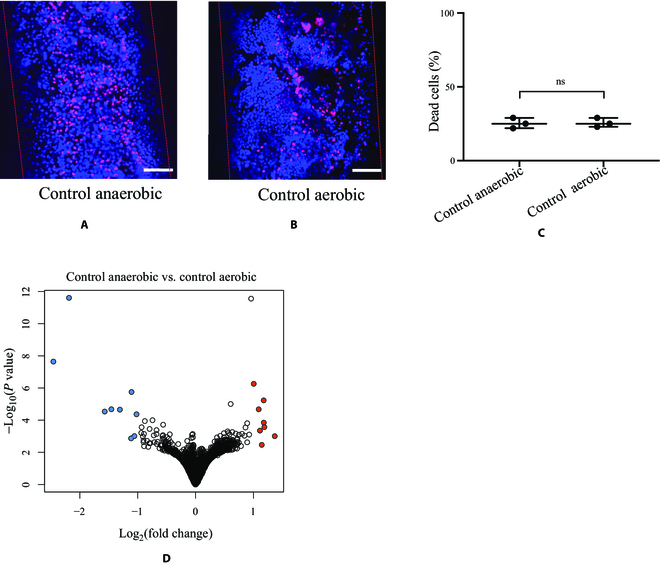
Cell gene expression under the anaerobic condition did not notably change compared to the aerobic condition in the gut 3D model. (A and B) Viability assays with live/dead staining of Caco-2 cells in the microchannel after culture under aerobic and anaerobic conditions. All the live cells were stained with blue fluorescence Hoechst 33342 solution, and the dead cells were stained with red PI fluorescence. (C) Quantification of dead cells under aerobic and anaerobic conditions. Data were analyzed by *t* test and represented as means ± standard deviation (SD) (ns, not significant). (D) Volcano plot of down-regulated (blue color) and up-regulated (red color) RNAs in Caco-2 cell culture in the 3D model under the anaerobic condition compared to the aerobic condition. The RNAs with a *P* value below 0.05 and fold change above 2 were indicated in colors. The scale bars are 100 μm.

After the 3D gut model was established over 21 d, we cultured the cells under the anaerobic condition for 12 h and evaluated the cell death rate and gene expressions for cells under the anaerobic condition. Then, we compared them to those under the aerobic condition. Data suggested that growth for 12 h under the anaerobic condition did not affect the viability of Caco-2 cells, with no significant difference in the death rates between the 2 conditions (Fig. 3A to C). RNA-seq analyses of the gene expressions revealed that 8 genes (*HBQ1*, *RNF186*, *PHGR1*, *ALDOB*, *RNU6ATAC*, *ANKRD37*, *TTR*, and *EFNA1*) were expressed significantly higher and 9 genes (*SLC7A11*, *CYP1A1*, *MT-TT*, *GCLM*, *MIRLET7A1HG*, *UGT1A1*, *FTX*, *N4BP2L2-IT2*, and *LINC01004*) were expressed significantly lower (fold change > 2, *P* < 0.05) in the anaerobic group compared to the aerobic group (Fig. 3D, Fig. S2, and Table S1). Notably, many of the altered genes are related to the oxygen response. For example, *HBQ1* (hemoglobin subunit theta 1) is associated with oxygen binding [32]. *ANKRD37* (ankyrin repeat domain 37) is a hypoxia-inducible factor 1 (HIF-1) target gene and plays an important role in the response to hypoxia [33]. *CYP1A1* (cytochrome P450 family 1 subfamily A member 1) is involved in the metabolism of numerous endogenous substrates by utilizing molecular oxygen and has been shown to be up-regulated by increased oxygen supplementation [34].

### Interactions between host cells and *Salmonella* under the aerobic condition in the 3D gut model

**Fig. 4.**
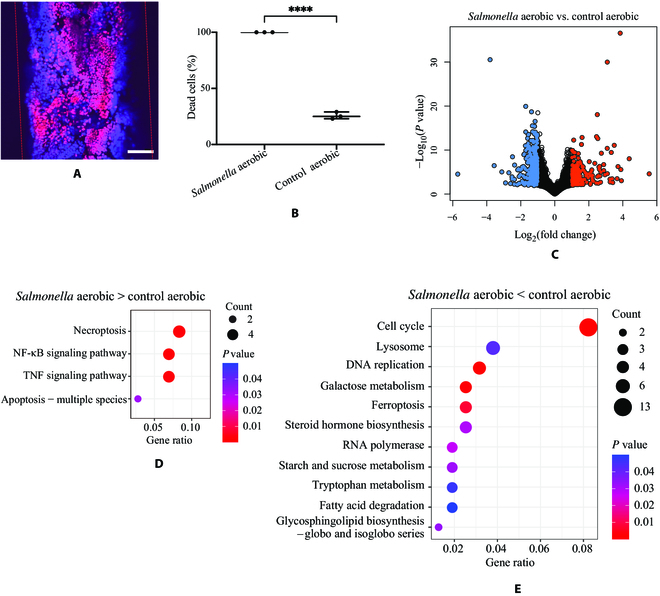
Interactions between host cells and *Salmonella* under the aerobic condition in the gut 3D model. (A) Viability assays with live/dead staining of Caco-2 cells in the microchannel after culture under the aerobic condition. All the live cells were stained with blue fluorescence Hoechst 33342 solution, and the dead cells were stained with red PI fluorescence. (B) Quantification of dead cells with and without *Salmonella* infection under the aerobic condition. Data between the 2 groups were analyzed by *t* test and represented as means ± SD (****P < 0.0001). (C) Volcano plot of up-regulated (red color) and down-regulated (blue color) RNAs in *Salmonella*-infected Caco-2 cell culture as compared to the uninfected cells in the 3D model under the aerobic condition. The RNAs with a *P* value below 0.05 and fold change above 2 were indicated in colors. (D and E) The KEGG pathway analysis of the differently expressed pathways that increased (D) and decreased (E) in *Salmonella*-infected cells compared to uninfected cells under the aerobic condition. Dot color represents the level of statistical significance. The number of RNAs in each pathway is indicated by the size of the dots. The scale bar is 100 μm. NF-κB, nuclear factor kappa B; TNF, tumor necrosis factor.

Having established the 3D gut model, as proof of concept, we first used it to investigate the interactions between host cells and *Salmonella*, which is one of the most common infectious pathogens invading host cells. Viability assays showed that almost all the cells were dead after 12 h of coculture with *Salmonella* (multiplicity of infection (MOI) around 10, Fig. 4A; MOI around 100, Fig. S3A). The cell death was significantly higher than that in the control cells without bacteria (Fig. 4B). However, when we used *Salmonella* culture supernatant or heat-killed *Salmonella*, no significantly increased cell death was observed, indicating that live bacteria would be essential for successful infection (Fig. S3B).

We continued studying the cell gene expressions under the aerobic condition with and without *Salmonella* infection. Many RNA coding components of the mitochondrial respiratory chain, such as *MT-CO1*, *MT-CYB*, *MT-ND2*, *MT-RNR2*, *MT-TE*, *MT-TI*, *MT-TP*, and *MT-TS1*, were highly elevated in the Caco-2 cells cocultured aerobically with *Salmonella* compared to the cells without infection (Fig. 4C, Fig. S4, and Table S2) [35]. Genes associated with inflammatory signaling, such as *CEBPB*, *RELB*, and *TNFAIP3*, were also expressed significantly higher in Caco-2 cells infected with *Salmonella* aerobically compared to the uninfected cells (Fig. S4 and Table S2) [36–38]. Kyoto Encyclopedia of Genes and Genomes (KEGG) analysis showed the enrichment of the necroptosis, nuclear factor kappa B (NF-κB) signaling pathway, and tumor necrosis factor (TNF) signaling pathway in the *Salmonella* aerobic group (Fig. 4D). In addition, the gene ontology (GO) enrichment analysis also revealed the enrichment of the inflammatory-associated pathways such as regulation of the inflammatory response pathway and antimicrobial humoral response pathway (Fig. S5A). In contrast, RNAs related to checkpoint signaling (e.g., *CDK5RAP3* and *CDC5L*) and meiotic cell cycle (e.g., *TOP2A*) were decreased in the cells infected by *Salmonella* compared to cells without infection under the aerobic condition (Fig. S4 and Table S2) [39–41]. KEGG and GO pathway analyses also suggested enrichment of cell cycle and DNA replication pathways in cells without *Salmonella* infection compared with the *Salmonella*-infected cells the under the aerobic condition (Fig. 4E and Fig. S5B).

### Interactions between host cells and *Salmonella* under the anaerobic condition in the 3D gut model

**Fig. 5.**
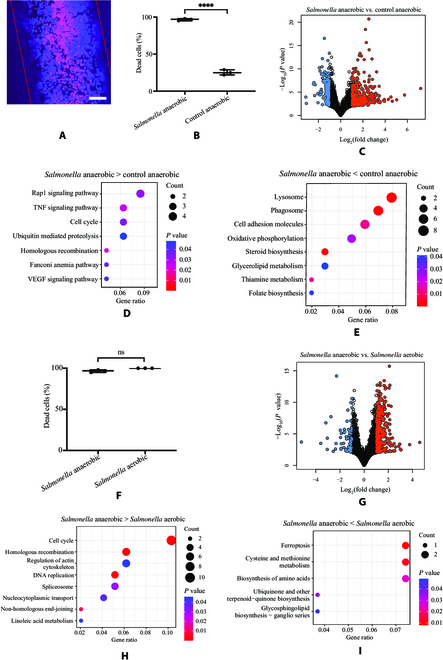
Interactions between host cells and *Salmonella* under the anaerobic condition in the gut 3D model. (A) Viability assays with live/dead staining of Caco-2 cells in the microchannel after culture with *Salmonella* under the anaerobic conditions. All the live cells were stained with blue fluorescence Hoechst 33342 solution, and the dead cells were stained with red PI fluorescence. The scale bar is 100 μm. (B) Quantification of the dead cells with and without *Salmonella* infection under the anaerobic condition. Statistical comparison between the 2 groups was carried out by *t* test, and data were represented as means ± SD (****P < 0.0001). (C) Volcano plot of up-regulated (red color) and down-regulated (blue color) RNAs in *Salmonella*-infected Caco-2 cell culture in the 3D model under the anaerobic condition as compared to the uninfected cells. The RNAs with a *P* value below 0.05 and fold change above 2 were indicated in colors. (D and E) The KEGG pathway analysis of the differently expressed pathways that increased (D) and decreased (E) in *Salmonella*-infected cells compared to uninfected cells under the anaerobic condition. (F) Quantification of dead cells after *Salmonella* infection under aerobic and anaerobic conditions. Data were compared by *t* test and represented as means ± SD. (G) Volcano plot of up-regulated (red color) and down-regulated (blue color) RNAs in Caco-2 cell culture after *Salmonella* infection in the 3D model under the anaerobic condition compared to aerobic condition. (H and I) The KEGG pathway analysis of the differently expressed pathways that increased (H) and decreased (I) in *Salmonella*-infected cells under the aerobic condition compared to the aerobic condition. Dot color represents the level of statistical significance. The number of RNAs in each pathway is indicated by the size of the dots. Rap 1, Ras–proximate-1; VEGF, vascular endothelial growth factor.

*Salmonella* cultured under the anaerobic condition showed significant inhibition on cell survival (Fig. 5A and B). Cell gene expressions under the anaerobic condition with and without *Salmonella* infection were also analyzed (Fig. 5C, Fig. S6, and Table S3). RNAs associated with the mitochondrial respiratory chain, such as *MT-CO1*, *MT-CYB*, and *MT-ND4*, were elevated in Caco-2 cells cultivated with *Salmonella* anaerobically compared to the uninfected cells [35]. RNAs involved in the apoptotic process such as *ATM* were increased in *Salmonella-*infected cells under the anaerobic condition, and RNAs related to cell metabolism, including *APOA2*, *APOH*, and *SOAT2* were decreased (Fig. S6 and Table S3) [42–44]. KEGG analysis showed enrichment of Ras–proximate-1 (Rap 1) signaling pathway and inflammatory TNF signaling pathway, which mediates cell survival and cell death-inducing signaling (Fig. 5D) [45]. Furthermore, less enrichment of lysosome, phagosome, and cell adhesion pathways in cells infected with *Salmonella* anaerobically compared to the uninfected cells was observed (Fig. 5E). GO pathway analysis indicated the enrichment of the positive regulation of nuclear division pathways and less enrichment of the protein–lipid remodeling and transport pathways in cells infected with *Salmonella* under the anaerobic condition compared to the uninfected cells (Fig. S7).

### Gene expressions of *Salmonella*-infected cells were different under aerobic and anaerobic conditions in the 3D gut model

To evaluate the difference between our anaerobic 3D gut model and the conventional aerobic model, we compared the cell gene expressions in the presence of *Salmonella* under the anaerobic condition with those under the aerobic condition. Although not significant, cell death with *Salmonella* under the anaerobic condition was a slightly less than the aerobic condition (Fig. 5F). We did not observe any bacterial gene expression difference in *Salmonella* under the aerobic condition in comparison to the anaerobic condition (Fig. S8)*.* However, cell gene expressions showed that 358 genes were significantly expressed higher and 68 genes were significantly expressed lower with *Salmonella* infection under the anaerobic condition than the aerobic condition (Fig. S9 and Table S4). Genes associated with meiotic cell cycle like *TOP2A* were significantly highly expressed in *Salmonella*-infected cells under the anaerobic condition compared to the aerobic condition (Fig. S9A and Table S4) [41]. In contrast, genes involved in cytokine activity such as *WNT9B* and *XCL1* were expressed lower in *Salmonella*-infected cells under the anaerobic condition than the aerobic condition (Fig. S9B and Table S4) [46,47]. KEGG pathway and GO pathway analyses demonstrated the enrichment of the cell cycle, DNA repair and replication pathways in *Salmonella*-infected cells under the anaerobic condition (Fig. 5H and Fig. S10). Pathways associated with amino acid biosynthesis and metabolism were enriched in *Salmonella*-infected cells under the aerobic condition than the anaerobic condition (Fig. 5I and Fig. S10).

### *Lactobacillus* interacts with host cells differently from *Salmonella* in the 3D gut model

**Fig. 6.**
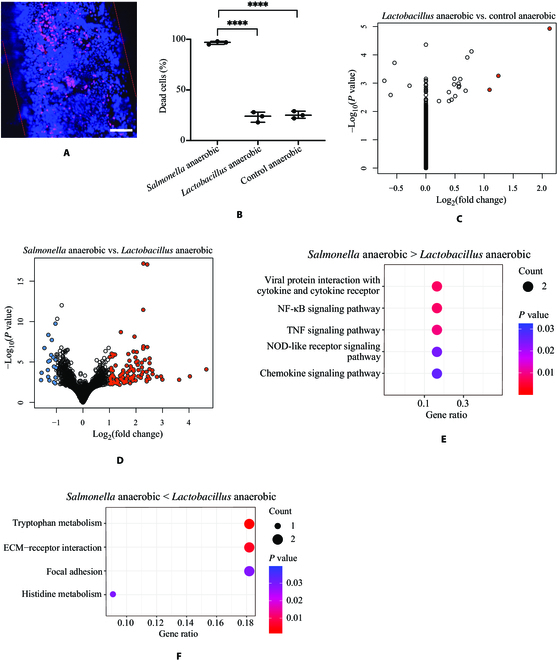
*Lactobacillus* interacts with host cells differently from *Salmonella* in the gut 3D model*.* (A) Viability assays with live/dead staining of Caco-2 cells in the microchannel after culture with Lactobacillus under the anaerobic conditions. All the live cells were stained with blue fluorescence Hoechst 33342 solution, and the dead cells were stained with red PI fluorescence. The scale bar is 100 μm. (B) Dead cell quantification of each culture condition and compared by one-way ANOVA with Dunnett’s multiple comparisons test. Data were represented as means ± SD (****P < 0.0001). (C) Volcano plot of up-regulated (red color) and down-regulated (blue color) RNAs in Caco-2 cells cocultured with *Lactobacillus* in the 3D model under the anaerobic condition compared to cells without *Lactobacillus*. (D) Volcano plot of up-regulated (red color) and down-regulated (blue color) RNAs in Caco-2 cells cocultured with *Salmonella* compared to cells with *Lactobacillus* in the 3D model under the anaerobic condition. (E and F) The KEGG pathway analysis of the differently expressed pathways that increased (E) and decreased (F) in *Salmonella*-infected cells compared to *Lactobacillus*-infected cells under anaerobic condition. Dot color represents the level of statistical significance. The number of RNAs in each pathway is indicated by the size of the dots. The RNAs with a *P* value below 0.05 and fold change above 2 were indicated in colors. NOD, nucleotide-binding oligomerization domain; ECM, extracellular matrix.

In addition to *Salmonella* as the pathogen, we evaluated *Lactobacillus* as a representative strain of the commensal gut microbiome. The Caco-2 cells cocultured with *Lactobacillus* (MOI around 10) for 12 h under the anaerobic condition showed no significant difference in cell death compared to the control group without bacteria (Fig. 6A and B). The RNA-seq analysis revealed that only 3 RNAs—*MYH7B*, *TMEM11-DT*, and *TCF4*—were up-regulated in the Caco-2 cells cocultured with *Lactobacillus* than cells alone in the 3D model under the anaerobic condition (Fig. 6C, Fig. S11, and Table S5). RNA-seq analysis between cells cocultured with *Salmonella* and *Lactobacillus* showed that many RNA expressions were altered (Fig. 6D, Fig. S12, and Table S6). For example, multiple genes associated with the mitochondrial respiratory chain, such as *MT-CO1*, *MT-CYB*, *MT-ND2*, and *MT-TI* were expressed higher in Caco-2 cells cocultured with *Salmonella*, while RNAs associated with peptidase regulator activity such as *SERPINC1* and *FN1* were higher in cells cocultured with *Lactobacillus* (Fig. S12) [48,49]. KEGG pathway analysis suggested that a number of inflammatory associated pathways, such as NF-κB signaling pathway, TNF signaling pathway, and nucleotide-binding oligomerization domain (NOD) pathway were enriched in the *Salmonella* coculture group compared to the *Lactobacillus* coculture group (Fig. 6E). In contrast, the tryptophan and histidine metabolism pathways and ECM–receptor interaction pathway were enriched in the *Lactobacillus* coculture group than the *Salmonella* coculture group (Fig. 6F). The GO pathway analysis also demonstrated that pathways related to chemokine and antimicrobial response as well as spindle checkpoint signaling were increased in the *Salmonella* coculture group (Fig. S13A). Moreover, pathways for regulation of body fluid, amino acid transport, and nutrient response were enriched in the *Lactobacillus* coculture group (Fig. S13B).

## Discussion

In this study, we seeded Caco-2 cells into 3D sacrificially printed GelMA hydrogel-embedded microchannels and successfully established a 3D gut epithelium model with high viability. We utilized the established 3D model to investigate host–microbe interactions under aerobic and anaerobic conditions. *Salmonella* killed essentially all the Caco-2 cells after 12 h of coculture under both aerobic and anaerobic conditions, while *Lactobacillus* did not affect the viability of the Caco-2 cells*.* RNA-seq revealed unnoticeable difference in Caco-2 cells without bacteria, cultured aerobically or anaerobically in the 3D model. We identified the enrichment of the pathways associated with the Rap1 signaling, TNF signaling, cell cycle, and homologous recombination in the Caco-2 cells cocultured with *Salmonella* under the anaerobic condition compared to the aerobic condition in the established 3D model. This difference highlighted the importance of studying the microbe–host interactions under the anaerobic condition in 3D models, which is arguably more closely related to the microenvironment within the human gut.

The crypt–villi structure of the gut enormously expands the intestinal surface area, which allows for enhanced nutrient uptake, barrier function, drug-metabolizing cytochrome P450 activity, and apical mucus secretion [50]. Several studies have reported the fabrication and usage of collagen-based scaffolds that mimic the 3D structure of intestinal epithelial [51–53]. It is suggested that Caco-2 is the most well-established cell model for mimicking the architecture and function of the small intestinal epithelial membrane, such as villi and crypt-like structures, tight junctions, and cell polarization [54]. The Caco-2 cells formed the preliminary 3D morphology in our model, which has provided a baseline for adding more cell types and generating more sophisticated human intestinal villi in the future.

With the development of the 3D sacrificially printed model, more applications for studying the gut microbiome were tested. 3D gut epithelial models have been used for molecular mechanism exploration of host–microbe interaction and antimicrobial drug screening of *Salmonella* infection [55–57]. Our study showed a great potential to use 3D model for investigation of the direct interaction between host cells and gut microbiome. We also observed the cell viability and gene expression differences under the anaerobic condition in comparison to the conventional aerobic condition in our 3D model.

We identified the enrichments of pathways associated with the inflammatory response in the *Salmonella* aerobic group compared to the control aerobic group. Specifically, the NF-κB signaling pathway and the TNF signaling pathway were overpresented in the *Salmonella* aerobic group. NF-κB plays an important role in defense against pathogens, and *Salmonella* lipopolysaccharide and flagellins have been reported to activate NF-κB signaling [58,59]. *Salmonella* has been reported to induce TNF receptor 1 signaling, regulating cell death and innate immune responses [60]. Our data under the aerobic condition supported the earlier findings and showed that our 3D sacrificially printed gut model was able to capture the important interactions between the host and the pathogen.

There is a strong need to keep oxygen at low concentrations (<0.5%) to support the survival of obligate anaerobes without injuring host cells, as most microbes in the gut are obligate anaerobes. To date, only limited reports have demonstrated 3D gut models cultured under the anaerobic conditions. A study coculturing the human intestinal epithelium with 11 well-characterized genera on-chip reported that species diversity of the anaerobic chips was significantly different from that of the aerobic chips [53]. The nanoporous membrane and the Transwell insert were also investigated to physically separate the intestinal bacterial species from the high oxygen environment [29–31]. However, the membranes separated the bacteria from the host cells, which might, in some cases, obstruct the interactions between the bacteria and host cells, especially for the bacteria that invade and inhabit cells. Our 3D gut epithelium model could be used for investigating direct host–microbe interactions under the anaerobic condition, which is essential for obligate anaerobic gut microbes.

Although no bacterial gene expression difference in *Salmonella* between aerobic and anaerobic conditions was observed, Caco-2 cells cocultured with *Salmonella* under aerobic and anaerobic conditions revealed a number of genes significantly differently expressed. Similar to what we found, studies using the microbial chamber observed a set of significantly differentially expressed genes in Caco-2 cells cocultured with the facultative anaerobe *Lactobacillus rhamnosus* GG (LGG) under the anaerobic conditions compare to the aerobic environment [31]. Cells cocultured with *Salmonella* under the anaerobic condition showed overpresentation of the pathways associated with cell cycle, homologous recombination, and DNA replication compared to cells cocultured with *Salmonella* under the aerobic condition. A high level of homologous recombination and remarkable cell cycle regulation is linked to a high proliferation rate and a longer S phase [61]. This suggests that the Caco-2 cells cocultured with *Salmonella* under the anaerobic condition might undergo S-phase arrest or initiate the cell repair procedures compared to the aerobic group. The different host cell responses to *Salmonella* under the anaerobic condition compared to the conventional aerobic condition demonstrated the great need for the consideration of the human gut anaerobic environment in future studies.

Nonetheless, this study has limitations. We cocultured the bacteria and the host cells for a comparatively short period under the anaerobic condition. Because *Salmonella* is very pathogenic and kills all the cells in 12 h, the direct bacteria–cell interactions could not be followed longer. However, the data from the gut commensal bacteria *Lactobacillus* in this study demonstrated the successful application of this established 3D gut model to assessing other commensal gut microbes for a longer follow-up time window to investigate the mechanism of host–microbe interactions. In addition, as a proof of concept, we inoculated only one cell type. More cell types in the human intestine epithelium, together with immune cells, could be added to the epithelial cells and mimic the sophisticated structure of the gut. Last, bacteria were added via single injection in our study, although including a flow system to explore the long-term interactions between the host and the microbiota might be of great value. The biggest challenge for the 3D gut microbiota models is to culture the stable anaerobic gut microbiota, especially together with a complex host system that contains multiple cell types and various interactions. Our proof-of-concept model paves the way for future clinical applications such as antimicrobial drug screening or antibiotic resistance testing with the consideration of the gut microbiota in 3D models.

In conclusion, we successfully established a 3D sacrificially printed gut epithelium model to coculture human cells with gut microbes under both aerobic and anaerobic conditions. In addition, the gene expression of both host cells and bacteria under aerobic and anaerobic conditions was compared with dual RNA-seq. Our demonstration provides a great model for further microbiome–host interactional studies and therapeutics screening at the 3D level under the anaerobic condition as more comprehensive gut–microbiome models are built.

## Materials and Methods

### Sacrificial printing

The 3D hydrogel construct was fabricated through the Allevi 2 bioprinter (3D Systems, USA) slightly modified from our previous reports [21,23,62]. Briefly, a layer of GelMA, synthesized according to our established protocol [63–65], was first deposited at the bottom of the PDMS mold with a dimension of 5 × 10 × 5 mm^3^ (*W* × *L* × *H*). The GelMA concentration at 5% was utilized in this work as it would allow proper mechanical properties to be produced that are suited for a variety of cell cultures [21,64,65]. An agarose microfiber (700 μm in diameter) was printed into the mold on the bottom GelMA hydrogel layer initially semi-crosslinked with UV light for 10 s, and then another layer of GelMA was filled on top and further crosslinked with UV light for 40 s. The scale of the microchannels used for sacrificial printing was based on our previously optimized conditions [21,23]. The scale of the microchannels used for sacrificial printing was based on our previously optimized conditions [21,23]. The size of the microchannels, although much smaller than the guts within the human system, would be a good fit for in vitro modeling and imaging purposes as in this work. Finally, the 3D GelMA hydrogel was retrieved from the PDMS holder with the agarose microfiber selectively removed to form the microchannel and stored in phosphate-buffered saline (PBS) at 4 °C for further use.

### Cell culture and seeding

Caco-2 epithelial cells derived from colon tissue with colorectal adenocarcinoma were used for establishing the 3D gut model. The cells were cultured in Dulbecco’s modified Eagle’s medium (DMEM) (Thermo Fisher, USA) with 10% fetal bovine serum (Thermo Fisher, USA) and 1% penicillin–streptomycin (10,000 units/ml and 10,000 μg/ml; Thermo Fisher, USA). Approximately 3.8 μl of cell suspension at a density of 5 × 10^6^ cells/ml was injected into the microchannel of each 3D hydrogel construct with a syringe needle (HSW HENKE-JECT, Germany). The 3D hydrogel with filled cells was incubated in a petri dish for 1 h without medium to assist the cells to attach onto the inner surface of the microchannel. The static cell culture inside the 3D hydrogel was then transferred into the wells of a 6-well plate filled with 2 ml of culture medium and incubated at 37 °C, 5% CO_2_.

### Cell staining

Cell morphology in the gut 3D model was observed at 3, 7, 14, and 21 d of culture. Briefly, the whole gel with cells inside was performed fixation with 4% paraformaldehyde (Electron Microscopy Sciences, USA), permeabilization with 0.1% Triton X-100 (Sigma-Aldrich, USA), and blocking with 5% bovine serum albumin (Thermo Fisher, USA) for 30 min at room temperature. Then, cytokeratin and nuclei were stained using monoclonal anti-pan cytokeratin (Sigma-Aldrich, USA) antibody and Hoechst 33342 (Thermo Fisher, USA), respectively, according to the manufacturer’s instructions. The pan cytokeratin antibody was diluted at a ratio of 1:50 (v/v) with 2% bovine serum albumin in PBS overnight (approximately 16 h). Hoechst 33342 solution was used at 10 μg/ml and incubated at 37 °C for 5 min. PBS washing steps were used 3 times with each time 5 min after fixation, permeabilization, blocking, and staining. The samples were finally washed once and observed for pan cytokeratin/nuclei staining.

Cell viability was tested at 14 and 21 d through staining with propidium iodide (PI) (10 μg/ml), which stains dead cells with red fluorescence, and Hoechst 33342 solution (10 μg/ml), which stains both dead cells and live cells with blue fluorescence. The gut 3D model was washed with PBS 3 times and then incubated with the PI and Hoechst dye mixture at room temperature in the dark for 30 min. All the images were obtained using the ZOE Fluorescent Cell Imager (bright-field; Bio-Rad, USA) and confocal microscope Zeiss LSM 800 Airyscan (fluorescence channels; ZEISS, Germany) at 10× controlled by ZEN blue 2.1 software.

### Bacterial culture

*Salmonella enterica* serovar Enteritidis PT4 (*Salmonella*) was cultured in Luria–Bertani broth aerobically at 37 °C. *Lactobacillus reuteri* DSM 17938 (*Lactobacillus*) was cultured in deMan–Rogosa–Sharpe (MRS) medium with the Oxoid AnaeroGen 2.5 L Sachet (Thermo Fisher, USA) into a 2.5-l BBL GasPak anaerobic system holding jar (BD, USA) to create an anaerobic environment. The Oxoid AnaeroGen 2.5 L Sachet reduces the oxygen content in the jar to below 1% within 30 min and around or below 0.1% after 2.5 h, producing an optimal atmospheric condition for the growth of intestinal anaerobic microorganisms.

### Bacteria and cell coculture condition

The live bacteria were spun down for 5 min at 13,000 rpm. The live bacteria pellet derived from centrifugation was resuspended in DMEM without fetal bovine serum and antibiotics. The heat-killed bacteria were obtained by heating the DMEM-resuspended bacteria at 95 °C for 30 min. The bacteria supernatant was obtained by filtering the overnight bacterial culture with a 0.2-μm filter.

For studying bacteria–cell interactions, the microchannel of the 3D hydrogel seeded with Caco-2 cells for 14 d was injected with 3.8 μl of 2.6 × 10^8^/ml of live bacteria (MOI around 100), heat-killed bacteria, and bacteria supernatant in the same amount of volume and concentration for cell viability analysis. The microchannel of 3D hydrogel seeded with Caco-2 cells for 21 d was also seeded with 3.8 μl of 2.6 × 10^7^/ml of live bacteria (MOI around 10) in DMEM for cell viability analysis and RNA-seq. The 2 ends of the microchannel were sealed with 2 plastic slices immediately after the injection of the bacteria. Then, the bacteria and cells were cultured in the wells of a 6-well plate at 37 °C for 12 h in an anaerobic environment created using the Oxoid AnaeroGen 2.5 L Sachet (Thermo Fisher, USA) in 2.5 l of BBL GasPak anaerobic system holding jar (BD, USA).

### RNA extraction

The 3D hydrogel containing cells and bacteria were bead-beaten with a ZR Bashing Bead Lysis tube (ZYMO Research, USA) with 600 μl of RLT Plus buffer in the RNeasy Plus Mini Kit (QIAGEN, Germany) to lyse both gram-positive and gram-negative bacterial strains completely. The setting for the bead beating was 1,600 rpm for 1 min with a 5-min pause. The cycles were repeated 5 times for a total of 5 min of bead beating with the 96 FastPrep shaker (MP Biomedicals, USA). The suspension was collected by centrifugation at 13,000 rpm for 1 min to avoid bead transfer. The RNA was further purified using the RNeasy Plus Mini Kit (QIAGEN, Germany) with a genomic DNA eliminator spin column to remove genomic DNA and with a RNeasy spin column to collect the total RNA. The purified RNA was stored at −80 °C before further RNA-seq.

### RNA-seq

Total RNA was subjected to quality and quantity control using a Eukaryotic Total RNA Pico assay on the Agilent 2100 Bioanalyzer instrument (Agilent, USA). To construct libraries suitable for Illumina sequencing, the Illumina Stranded Total RNA Prep, Ligation with Ribo-Zero Plus was used (Illumina, USA). First, ribosomal RNA was depleted from 20 ng of total RNA using a chemical depletion method. Next, RNA was denatured, fragmented, and transformed into single-stranded cDNA with random hexamer priming followed by second-strand synthesis. Blunt-end fragments were created with a combination of fill-in reactions and exonuclease activity. Then, an A-base was added to the blunt ends to prevent them from ligating to each other during the following pre-index anchor ligation. Finally, polymerase chain reaction was used to selectively amplify the anchor-ligated DNA fragments and add indexes and primer sequences for cluster generation. The indexed libraries were then normalized and combined, and the pool was sequenced on the Illumina Nextseq 2000 P2 flow cell for a 116-bp paired-end sequencing run, generating 58-bp pair-end reads.

### RNA-seq analysis

Basecalling and demultiplexing were performed using Illumina bcl2fastq (v2.20), and adapter trimming was performed using CutAdapt (v3.5). Sequence data quality was assessed using FastQC (v0.11.8). To find the best genome match, the BBSplit tool from BBMap (v38.41) was used to first split reads between human and bacteria and, subsequently, between *Salmonella* and *Lactobacillus*. Read alignment to the respective genome was performed using STAR (v2.6.1d). Gene counts were estimated using featureCounts (v1.5.1). Genomic reference sequences were collected from Ensembl (human) and RefSeq (*Salmonella*, GCF_015240635.1 and *Lactobacillus*, GCA_000159455.2). Count data were imported to Bioconductor package DESeq2 (v1.34) and tested for differential expression using shrink log_2_ fold change, estimating with apeglm method and analyzing the significance with Wald tests. Log fold change shrinkage (apeglm method) and *P* values of Wald test were applied to compare the gene expression. The expression of gene with annotated gene names between 2 groups were displayed with volcano plot. The top 50 up-regulated and top 50 down-regulated genes were shown in heatmap (*P* < 0.05, fold change > 2). The differentially expressed genes were analyzed with GO enrichment analysis. The top 30 significant represented biological process-associated GO pathways (*P* < 0.05) were displayed with tree plot. The enriched pathways of the differentially expressed genes (*P* < 0.05) were also analyzed through KEGG analysis. The minimal size of the RNA set for KEGG pathway analysis was 10 RNAs, and the maximal size of each RNA set for analysis was 500 RNAs. The KEGG pathway involved in metabolism, genetic information processing, environmental information processing, and cellular processes were shown in the dot plot.

### Statistical analyses

The difference of cell viability between 2 groups were compared by *t* test. The difference of cell viability between 3 groups were compared by one-way analysis of variance (ANOVA) with Dunnett’s multiple comparisons test. The difference of RNA levels between 2 groups were compared by Wald test and fold change with DESeq2. The differentially expressed genes were chosen with a fold change greater than 2 and a *P* value lower than 0.05.

## Data Availability

All the data generated or analyzed for this study are included in this paper. The RNA-seq reads are available in the Sequence Read Archive (SRA) of the National Center for Biotechnology Information under accession project number PRJNA888875.

## Supplementary Materials

Supplementary 1 Fig. S1. The cell number of the Caco-2 cells of the cross-sectional slides on day 21 in the gut 3D model. Fig. S2. Heatmap of the significantly differentially expressed genes between Caco-2 cells cultured under aerobic and anaerobic conditions. Fig. S3. Viability assays of Caco-2 cells cocultured with *Salmonella* and supernatant under the aerobic condition. Fig. S4. Heatmap of the significantly differentially expressed genes between *Salmonella*-infected Caco-2 cells and the uninfected cells in the 3D model under the aerobic condition. Fig. S5. The top GO pathways enriched in *Salmonella*-infected Caco-2 cells and the uninfected cells in the 3D model under the aerobic condition. Fig. S6. Heatmap of the significantly differentially expressed genes between *Salmonella*-infected Caco-2 cells and the uninfected cells in the 3D model under the anaerobic condition. Fig. S7. The top GO pathways enriched in *Salmonella*-infected Caco-2 cells and the uninfected cells in the 3D model under the anaerobic condition. Fig. S8. Volcano plot of *Salmonella* RNAs in the 3D model under the anaerobic condition compared to the aerobic condition. Fig. S9. Heatmap of the significantly differentially expressed genes between Caco-2 cells cocultured with *Salmonella* under aerobic and anaerobic conditions in the 3D model. Fig. S10. The top GO pathways enriched in Caco-2 cells cocultured with *Salmonella* under aerobic and anaerobic conditions in the 3D model. Fig. S11. Heatmap of the 3 significantly differentially expressed RNA between Caco-2 cells cocultured with *Lactobacillus* in the 3D model under the anaerobic condition and cells without *Lactobacillus*. Fig. S12. Heatmap of the significantly differentially expressed genes between Caco-2 cells cocultured with *Salmonella* and with *Lactobacillus* in the 3D model under the anaerobic condition. Fig. S13. The top GO pathways enriched in Caco-2 cells cocultured with *Salmonella* and with *Lactobacillus* in the 3D model under the anaerobic condition.

Supplementary 2 Table S1. The gene expression data comparison between cells cultured under aerobic and anaerobic conditions. Table S2. The gene expression data comparison of the cells with and without *Salmonella* infection under the aerobic condition in the gut 3D model. Table S3. The gene expression data comparison of the cells with and without *Salmonella* infection under the anaerobic condition in the gut 3D model. Table S4. The gene expression data comparison of cells under aerobic and anaerobic conditions with *Salmonella* infection in the gut 3D model. Table S5. The gene expression data comparison of the cells with and without *Lactobacillus* coculture under the anaerobic condition in the gut 3D model. Table S6. The gene expression data comparison of the cells with *Salmonella* coculture and with *Lactobacillus* coculture under the anaerobic condition in the gut 3D model.
