# Supplementary material for: A 3D Bioprinted Gut Anaerobic Model for Studying Bacteria–Host Interactions
Source: Research (Wash D C). 2023 Feb 27;6:0058. doi: 10.34133/research.0058 (PMC10076011; doi:10.34133/research.0058)

Supplementary Figure 1

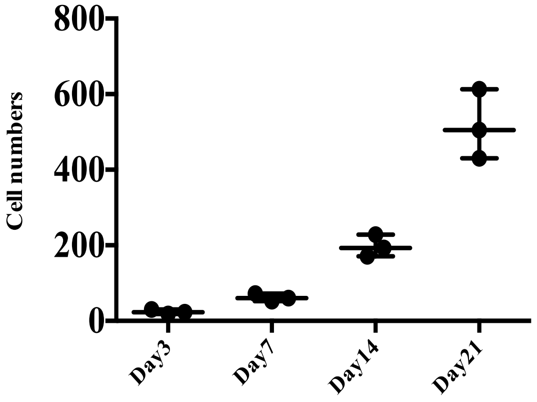

Supplementary Figure 2

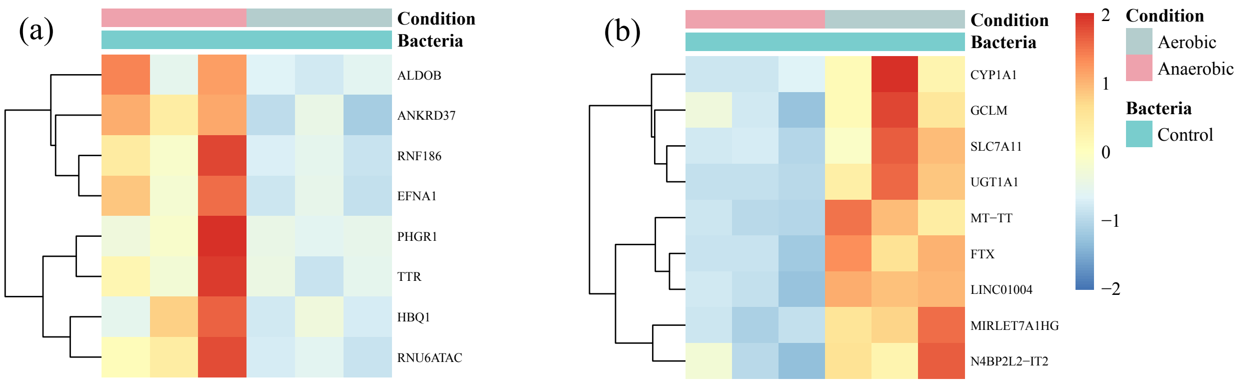

Supplementary Figure 3

(a)

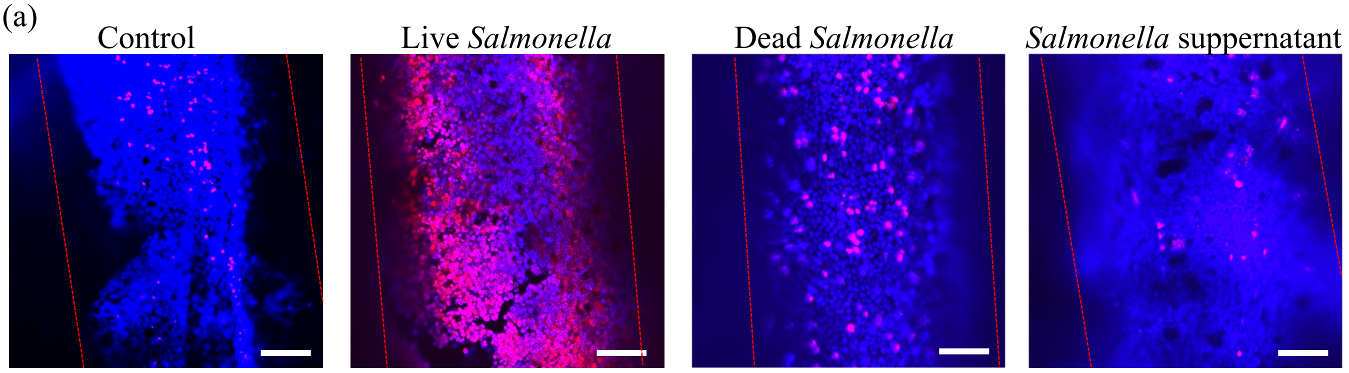

(b)

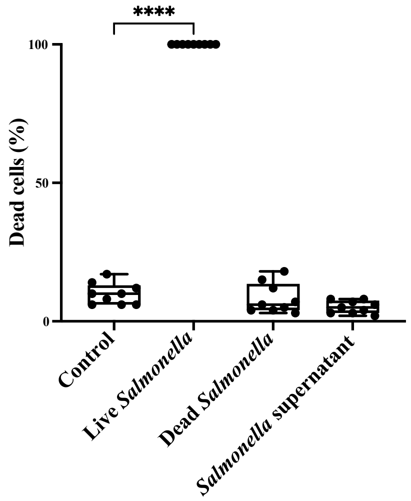

Supplementary Figure 4

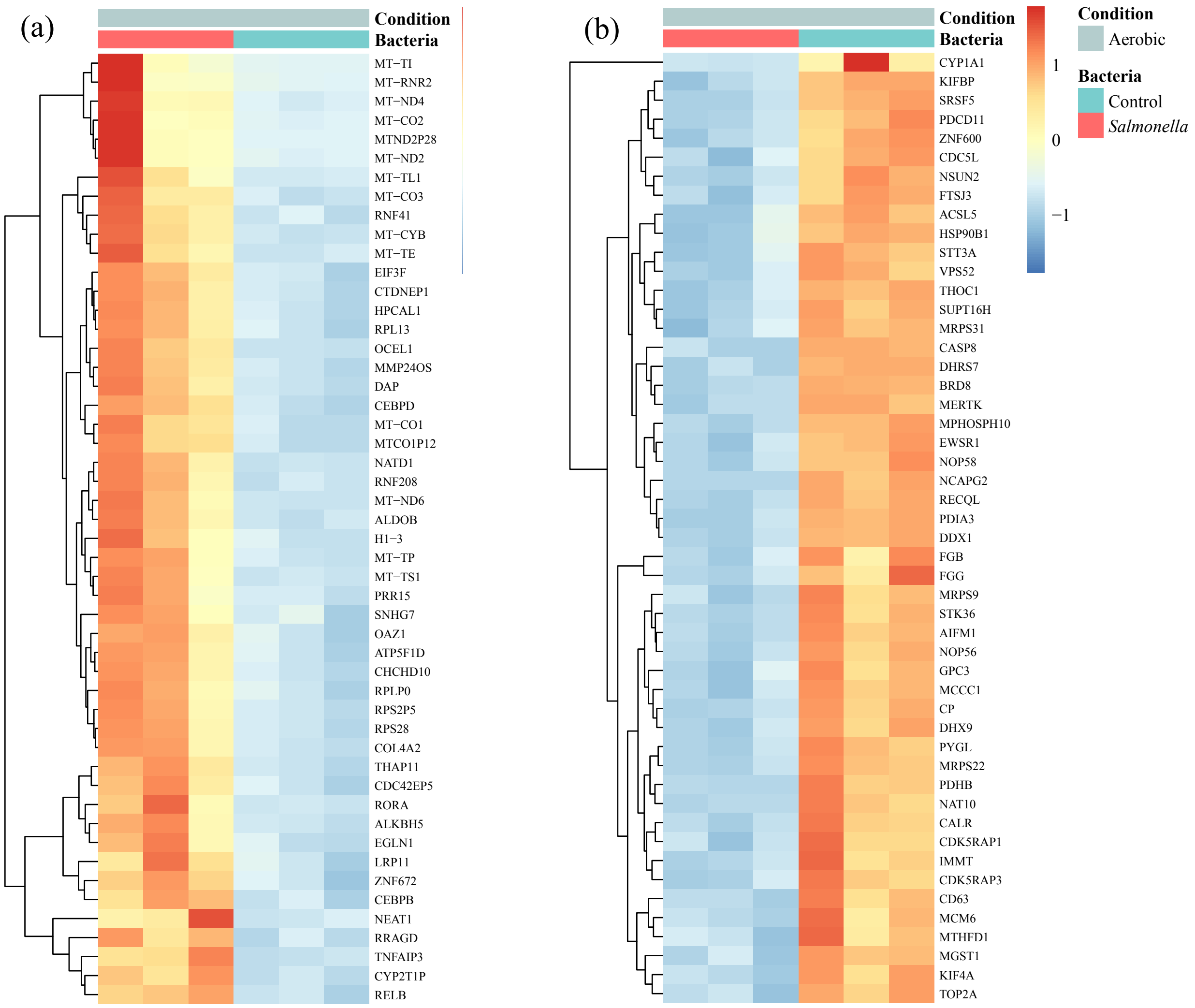

Supplementary Figure 5

(a)

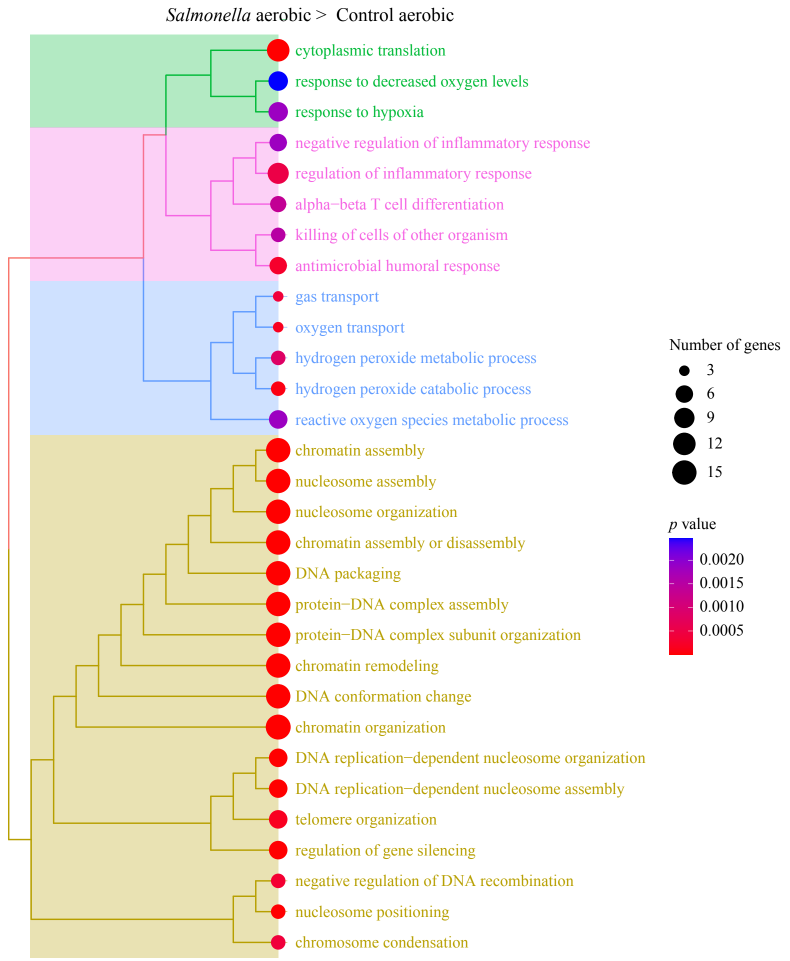

(b)

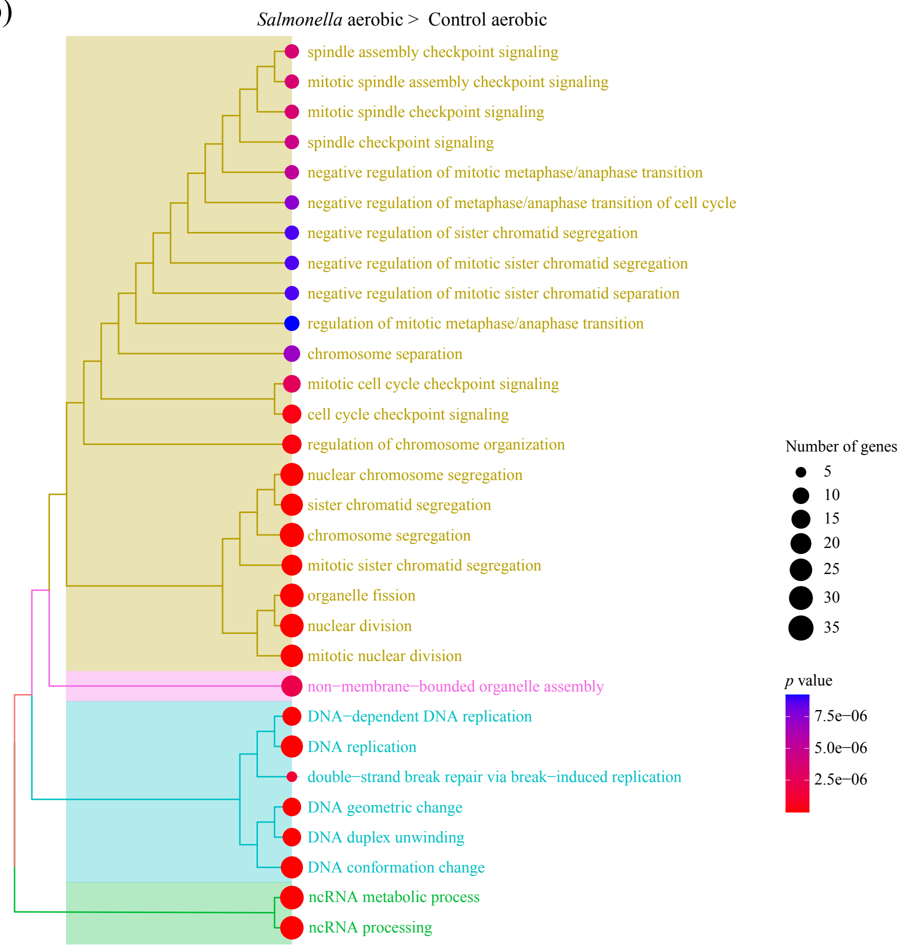

Supplementary Figure 6

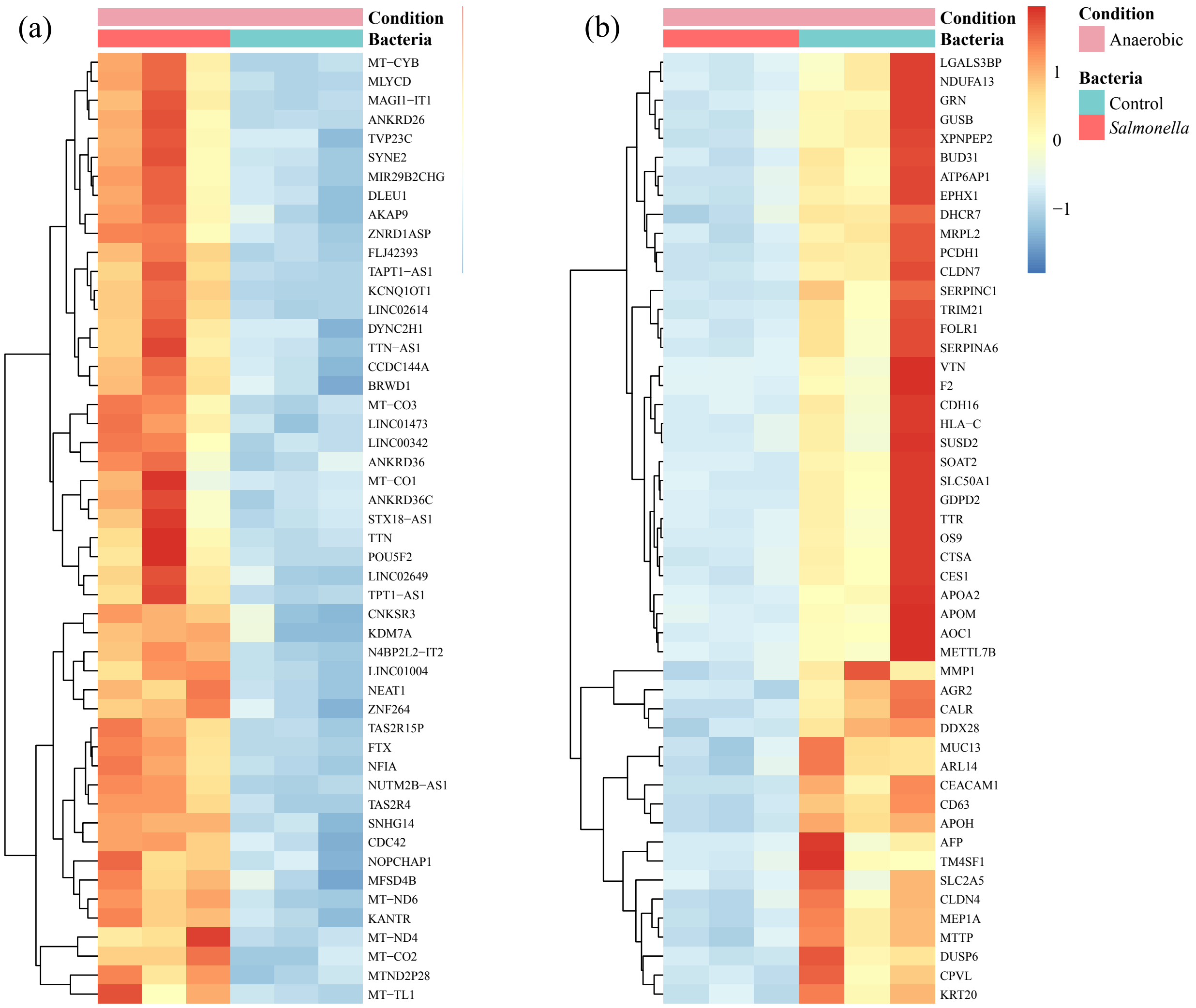

Supplementary Figure 7

(a)

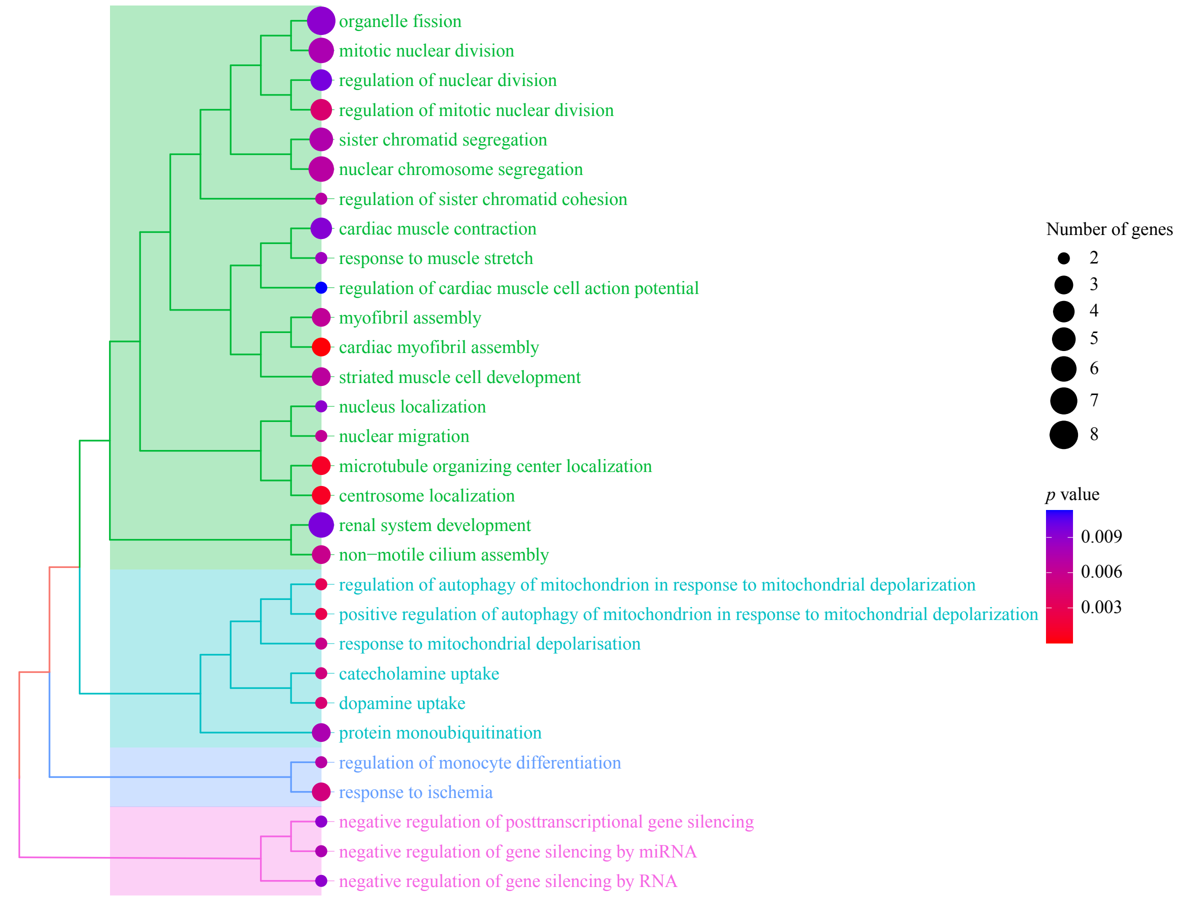

(b)

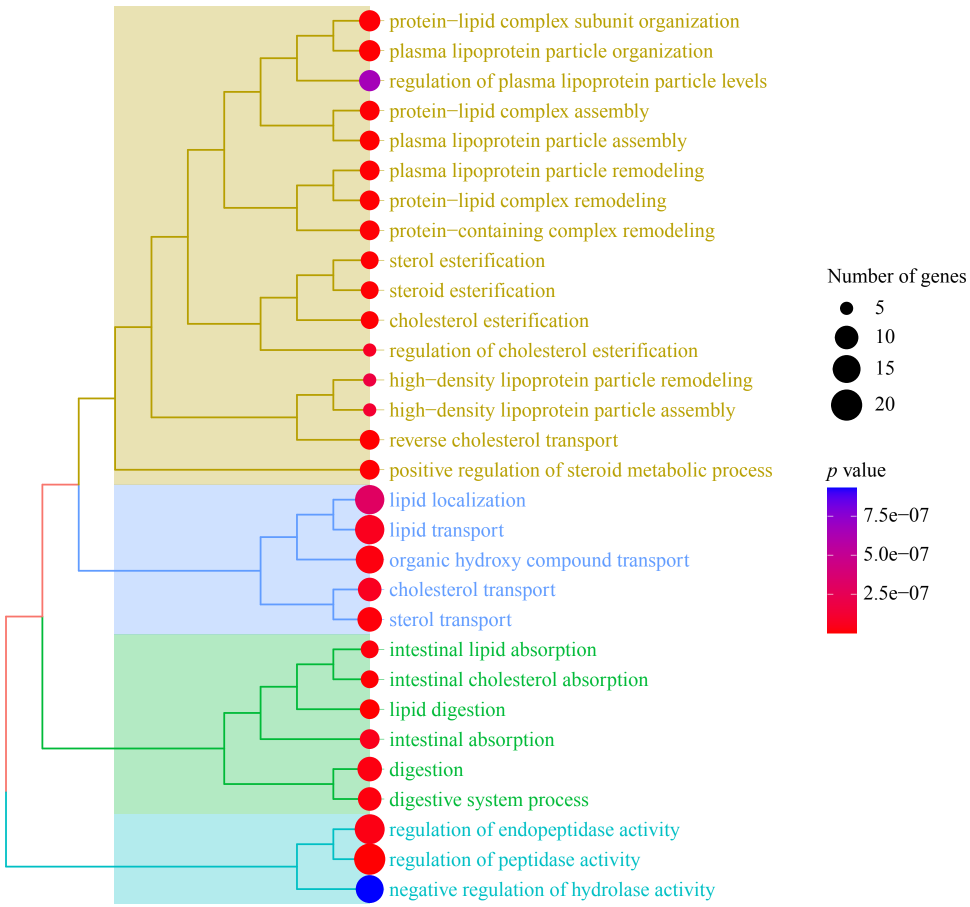

Supplementary Figure 8

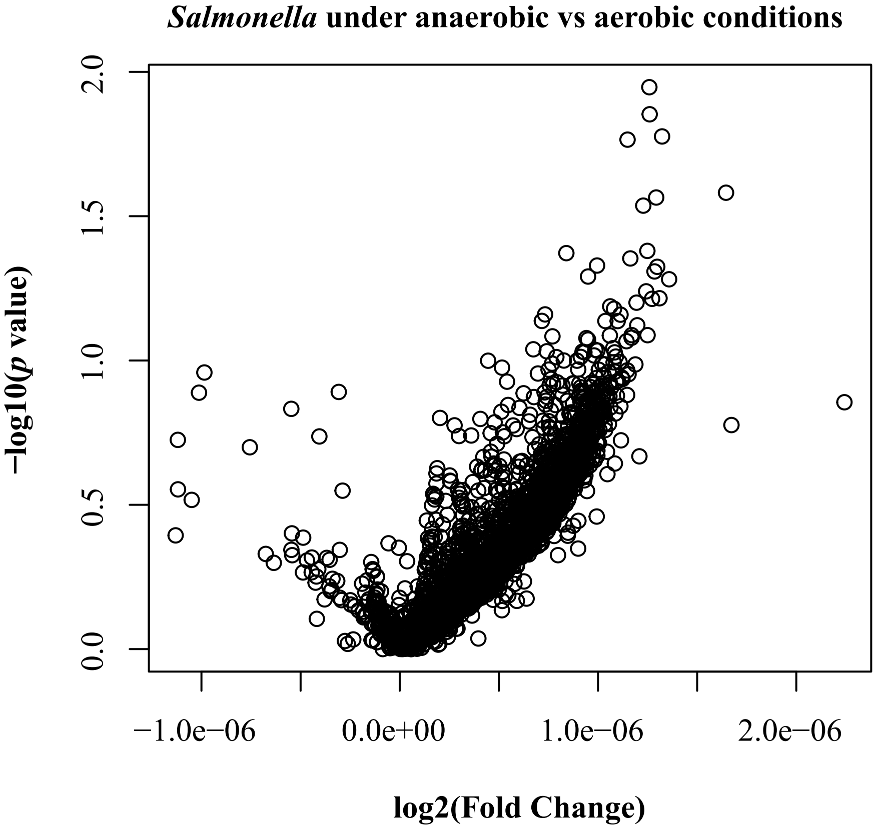

Supplementary Figure 9

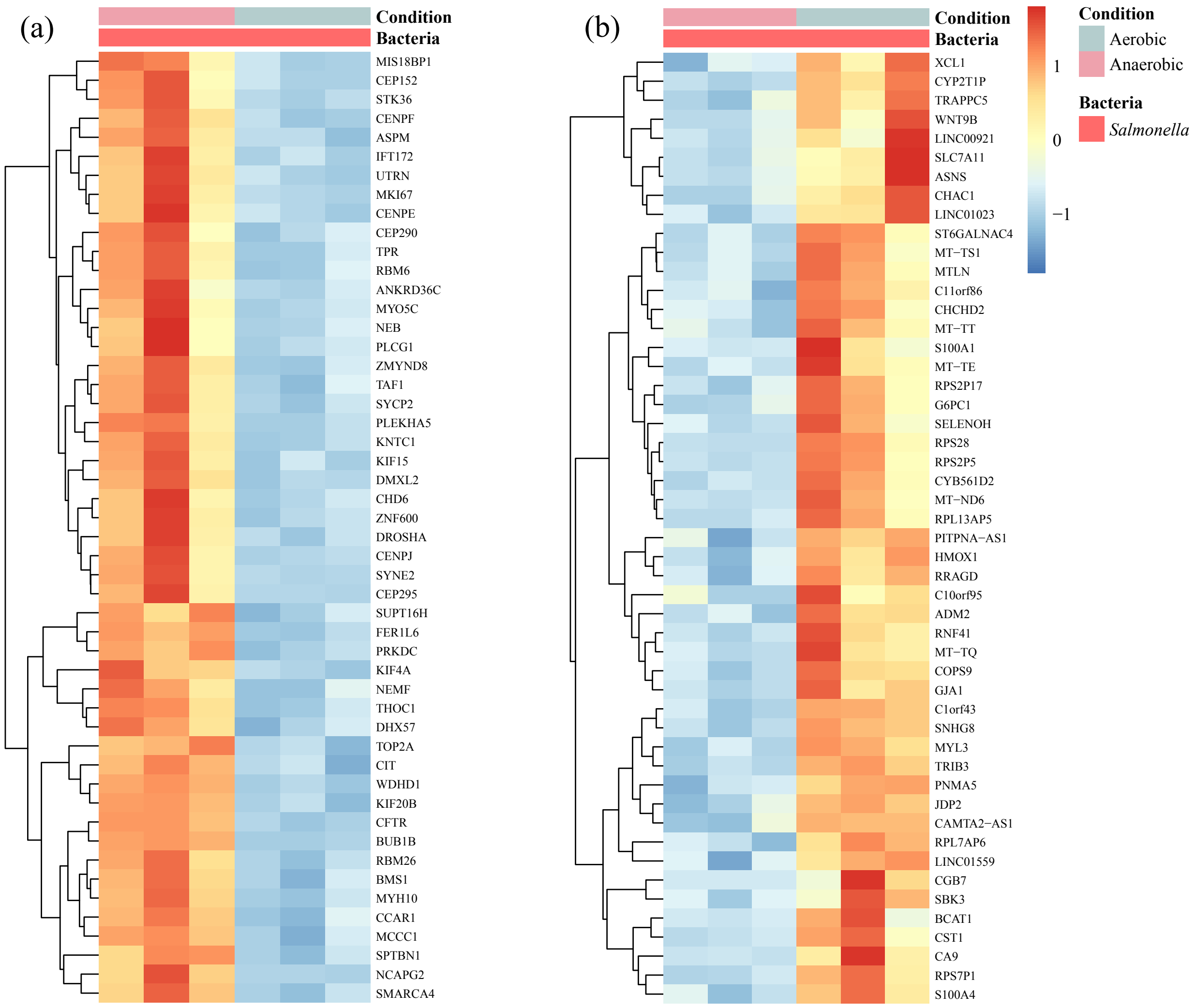

Supplementary Figure 10

(a)

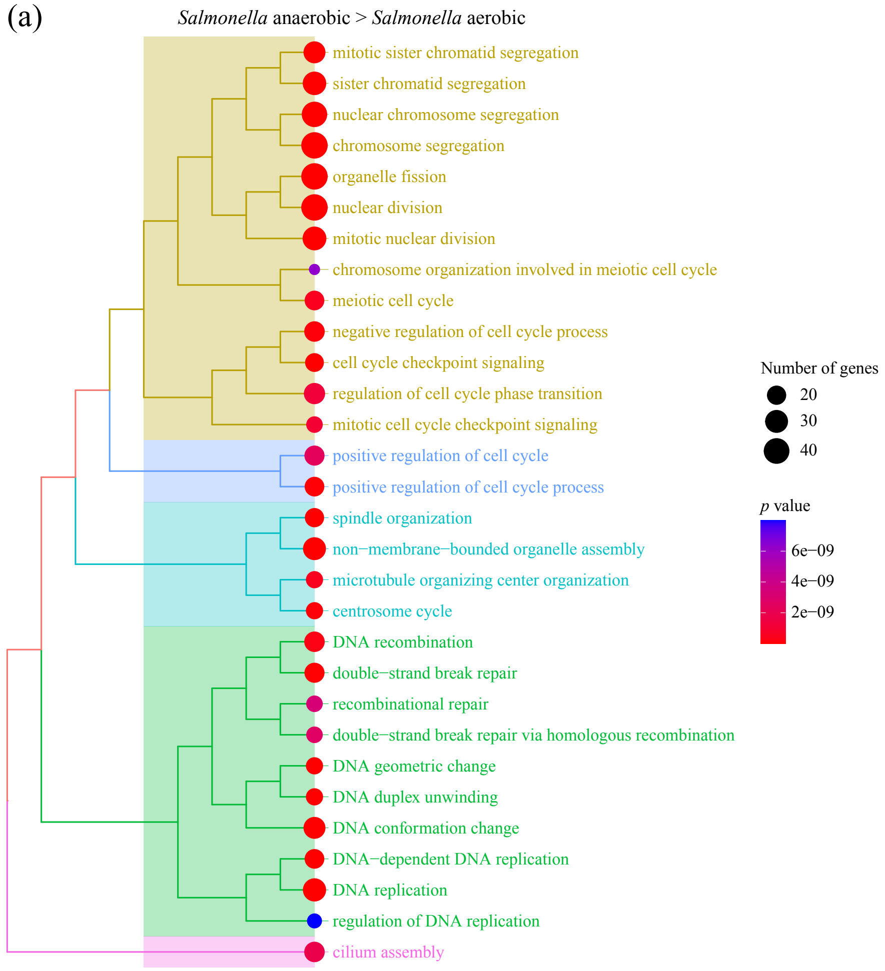

(b)

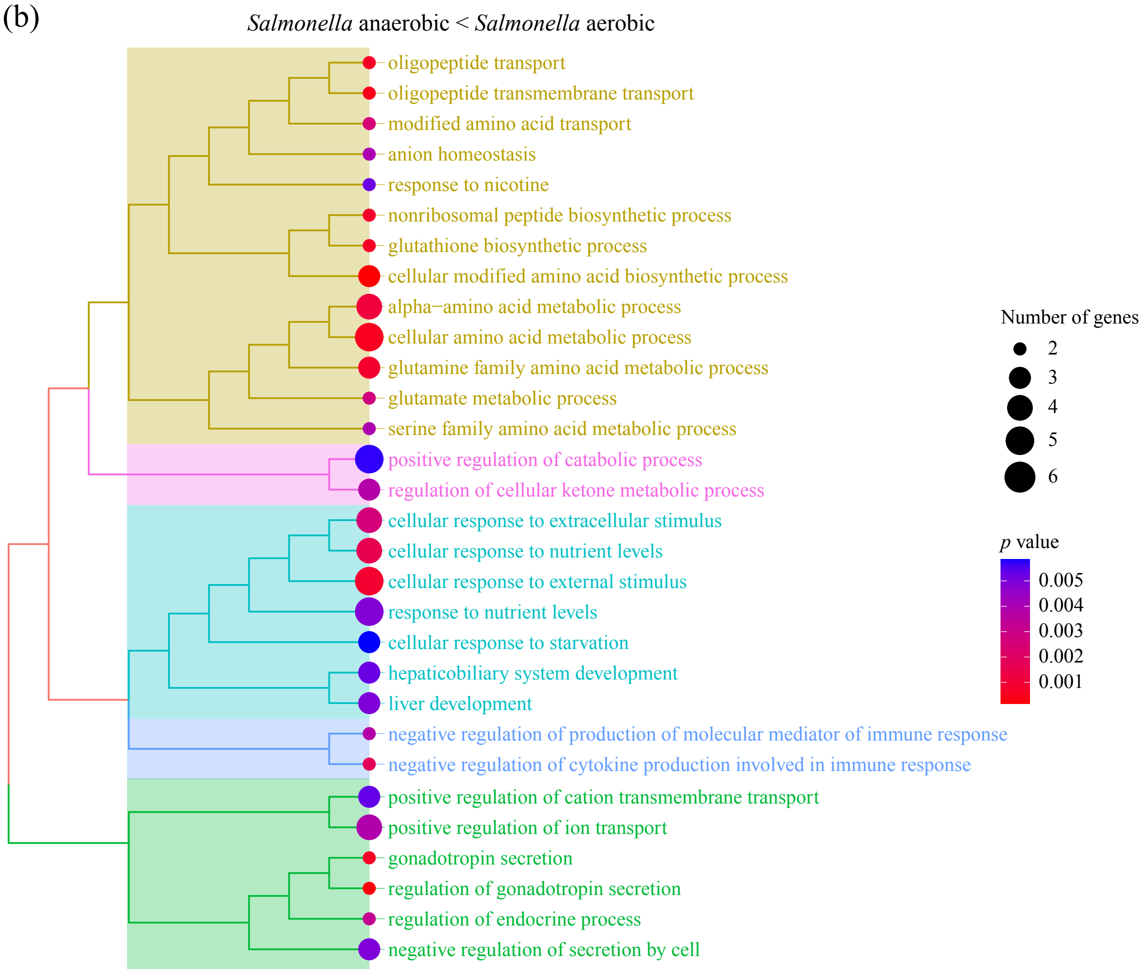

Supplementary Figure 11

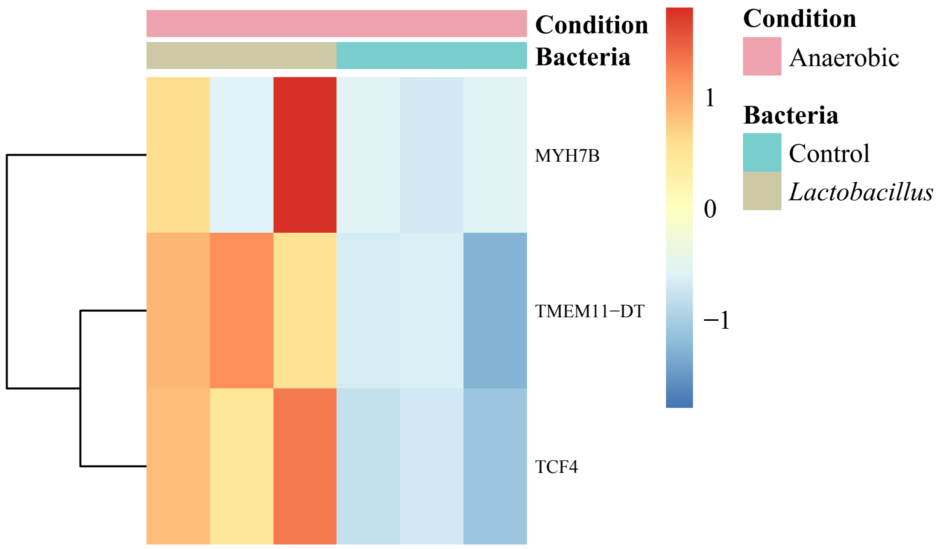

Supplementary Figure 12

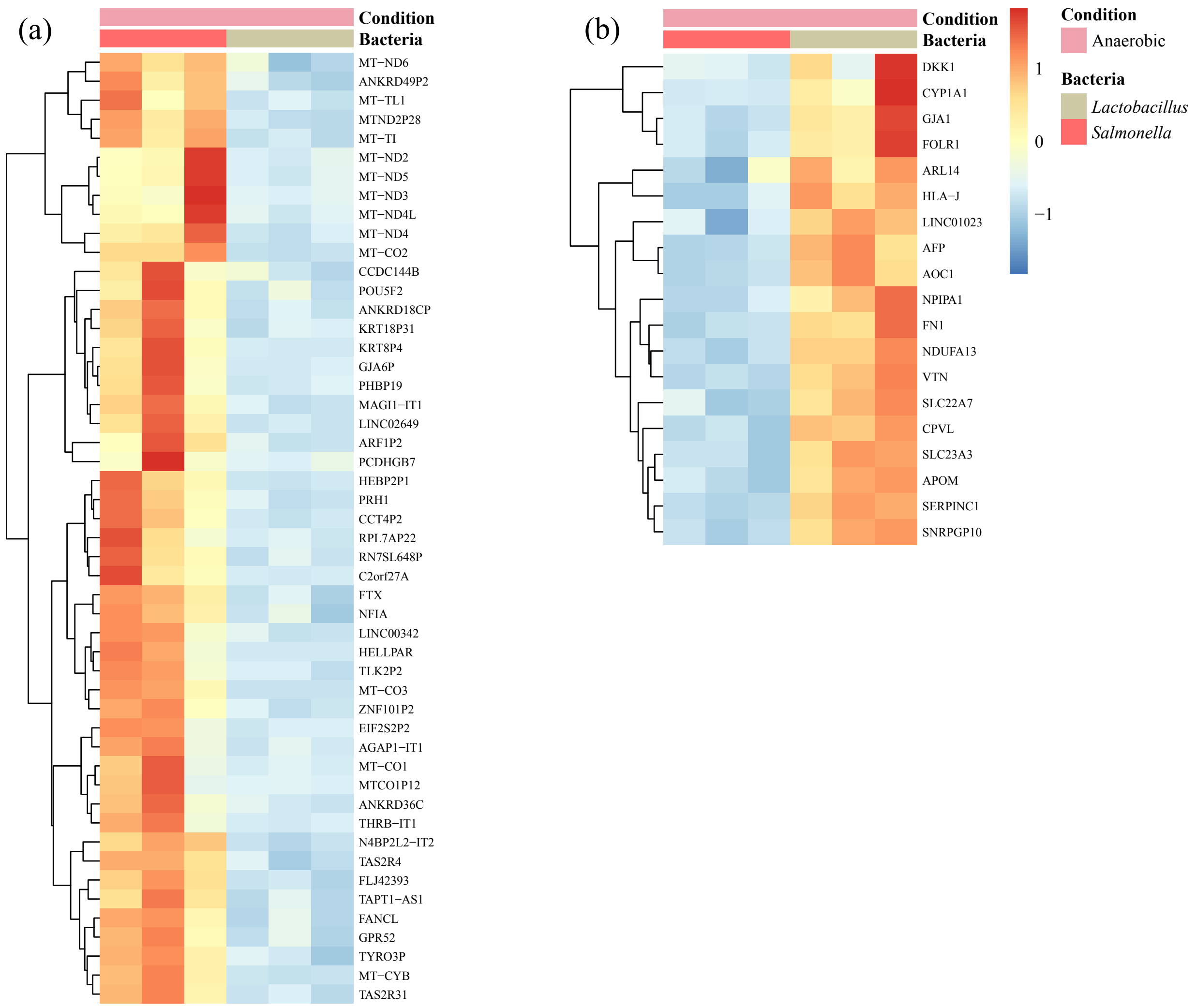

Supplementary Figure 13

(a)

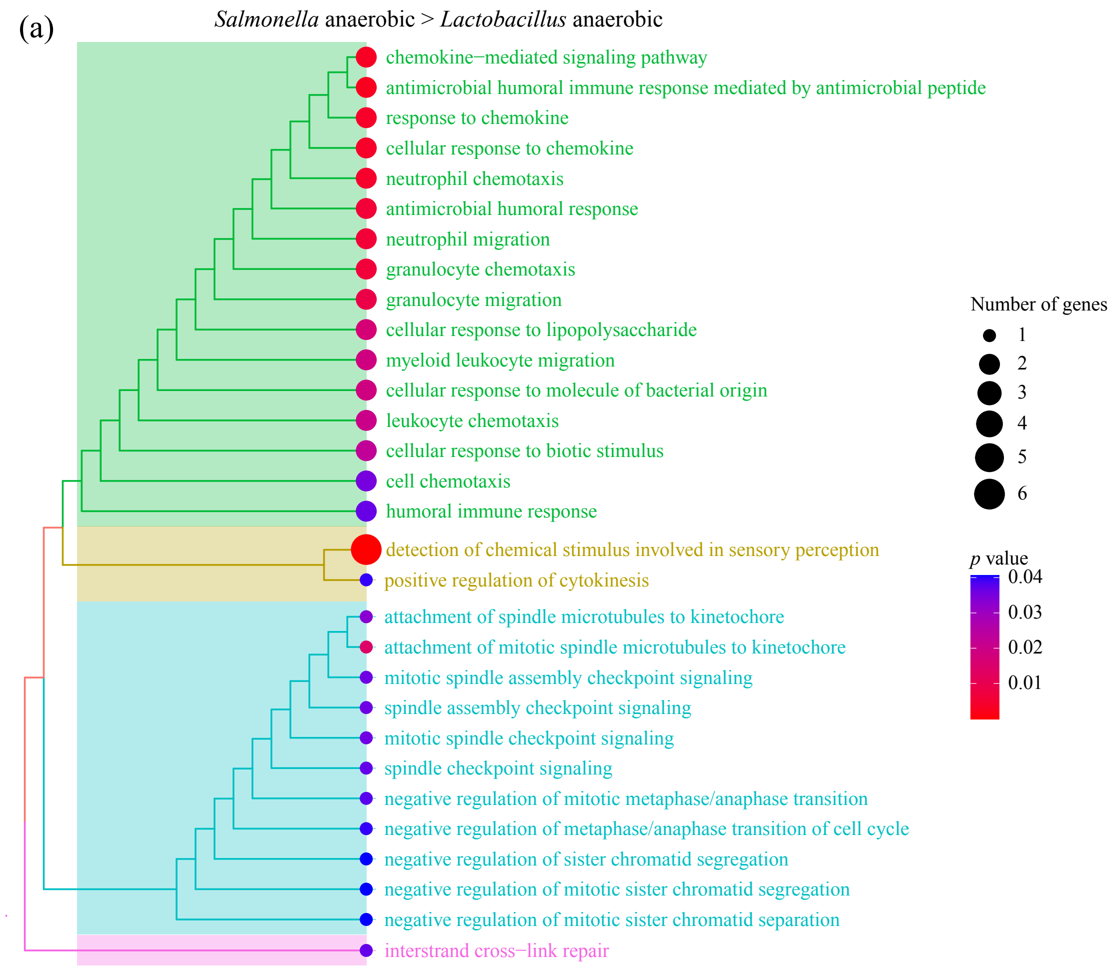

(b)

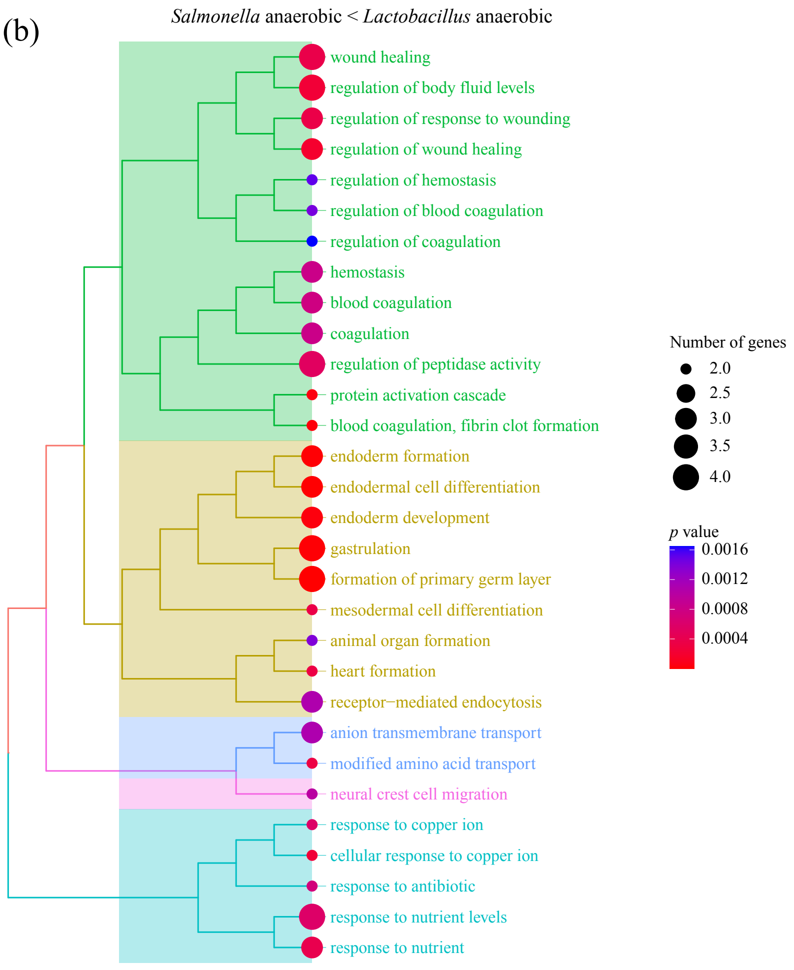

Supplement: Supplementary 1 — Fig. S1. The cell number of the Caco-2 cells of the cross-sectional slides on day 21 in the gut 3D model. Fig. S2. Heatmap of the significantly differentially expressed genes between Caco-2 cells cultured under aerobic and anaerobic conditions. Fig. S3. Viability assays of Caco-2 cells cocultured with Salmonella and supernatant under the aerobic condition. Fig. S4. Heatmap of the significantly differentially expressed genes between Salmonella-infected Caco-2 cells and the uninfected cells in the 3D model under the aerobic condition. Fig. S5. The top GO pathways enriched in Salmonella-infected Caco-2 cells and the uninfected cells in the 3D model under the aerobic condition. Fig. S6. Heatmap of the significantly differentially expressed genes between Salmonella-infected Caco-2 cells and the uninfected cells in the 3D model under the anaerobic condition. Fig. S7. The top GO pathways enriched in Salmonella-infected Caco-2 cells and the uninfected cells in the 3D model under the anaerobic condition. Fig. S8. Volcano plot of Salmonella RNAs in the 3D model under the anaerobic condition compared to the aerobic condition. Fig. S9. Heatmap of the significantly differentially expressed genes between Caco-2 cells cocultured with Salmonella under aerobic and anaerobic conditions in the 3D model. Fig. S10. The top GO pathways enriched in Caco-2 cells cocultured with Salmonella under aerobic and anaerobic conditions in the 3D model. Fig. S11. Heatmap of the 3 significantly differentially expressed RNA between Caco-2 cells cocultured with Lactobacillus in the 3D model under the anaerobic condition and cells without Lactobacillus. Fig. S12. Heatmap of the significantly differentially expressed genes between Caco-2 cells cocultured with Salmonella and with Lactobacillus in the 3D model under the anaerobic condition. Fig. S13. The top GO pathways enriched in Caco-2 cells cocultured with Salmonella and with Lactobacillus in the 3D model under the anaerobic condition. [file research.0058.f1.pdf]
